# Validity and Reliability of Mobile Applications for Assessing Strength, Power, Velocity, and Change-of-Direction: A Systematic Review

**DOI:** 10.3390/s21082623

**Published:** 2021-04-08

**Authors:** Rui Silva, Markel Rico-González, Ricardo Lima, Zeki Akyildiz, José Pino-Ortega, Filipe Manuel Clemente

**Affiliations:** 1 Instituto Politécnico de Viana do Castelo, Escola Superior Desporto e Lazer, Rua Escola Industrial e Comercial de Nun’Álvares, 4900-347 Viana do Castelo, Portugal; ricardo.lima@esdl.ipvc.pt (R.L.); filipe.clemente5@gmail.com (F.M.C.); 2 Department of Physical Education and Sport, University of the Basque Country, UPV-EHU, Lasarte 71, 01007 Vitoria-Gasteiz, Spain; markeluniv@gmail.com; 3 BIOVETMED & SPORTSCI Research Group, Department of Physical Activity and Sport, Faculty of Sport Sciences, University of Murcia, San Javier, 30100 Murcia, Spain; josepinoortega@um.es; 4 The Research Centre in Sports Sciences, Health Sciences and Human Development (CIDESD), 5001-801 Vila Real, Portugal; 5 Sports Science Department, Gazi University, Teknikokullar, Ankara 06500, Turkey; zekiakyldz@hotmail.com; 6 Faculty of Sports Sciences, University of Murcia, 30720 San Javier, Spain; 7 Instituto de Telecomunicações, Delegação da Covilhã, 1049-001 Lisboa, Portugal

**Keywords:** sports technology, smartphone, accuracy, precision, athletic performance, fitness

## Abstract

This systematic review aimed to (1) identify and summarize studies that have examined the validity of apps for measuring human strength, power, velocity, and change-of-direction, and (2) identify and summarize studies that have examined the reliability of apps for measuring human strength, power, velocity, and change-of-direction. A systematic review of Cochrane Library, EBSCO, PubMed, Scielo, Scopus, SPORTDiscus, and Web of Science databases was performed, according to the Preferred Reporting Items for Systematic Reviews and Meta-Analyses (PRISMA) guidelines. From the 435 studies initially identified, 23 were fully reviewed, and their outcome measures were extracted and analyzed. In total, 11 mobile applications were analyzed and summarized for their validity and reliability to test movement velocity, movement time, movement displacement, power output, and workload. The present systematic review revealed that the tested apps are valid and reliable for measuring bar movement velocity during lower and upper body resistance exercises; however, systematic bias was detected with heavier loads.

## 1. Introduction

Performance and fitness assessments are common processes related to the individualization of training [1,2,3,4]. Different physical qualities can be screened in a fitness assessment battery [5,6,7,8]. The most typical ones are related to neuromuscular-related qualities, with strength and power [9,10,11], velocity [12,13], and change-of-direction [14,15] being the most prevalent. Typically, strength is assessed considering the lifted load or the velocity at which the load is lifted [16,17,18]. In the case of neuromuscular power (or impulse), not only is weightlifting monitored but so are other movements for which height, flight time, or contact time are considered (e.g., jumping) [19,20]. For assessing strength and power, dynamometers [21,22], linear transducers [23,24], optoelectronic systems [25,26], or force plates [27,28] are usually used to measure the movements and their intensity [29]. In the case of running velocity (sprinting) or change-of-direction tests, the time of movement between two points is usually the common outcome [30]. Photocells and timers are considered gold standard instruments for measuring this parameter [31,32].

Such assessments are typically performed in a laboratory or field-based context. However, the cost of some gold standard instruments can prevent the massification of performance or fitness assessments by strength and conditioning coaches across different economic contexts and practical scenarios [33]. However, continuous improvements in the sensors and tools included in mobile devices have made it possible to develop mobile applications (apps) that serve as alternatives to gold standard instruments [34]. In fact, the development of apps for sports sciences is ongoing, making it possible to provide a wide range of opportunities to those with limited access to expensive or gold standard instruments [35].

As mentioned, typical outcomes related to strength and power, velocity, and change-of-direction actions have focused on the velocity, time, or the displacement of a movement [36]. These main outcomes are, in a sense, able to take measurements using image-based or video-based analyses incorporated into smartphone cameras [37,38,39]. Though they are not automatic, a wide range of apps have simple and user-friendly processes for collecting and treating data. However, this does not dismiss the need for a human operator to perform the operations, and this might increase the risk of inaccuracy or imprecision. Therefore, a growing number of original studies have tested the validity and reliability of these sports sciences apps [40,41], aiming to determine their capacity to be used for performance and fitness assessments.

The use of mobile applications has a wide range. Mobile apps are frequently used by sports scientists, strength and conditioning coaches, and practitioners to measure physical conditioning [42]. The inaccessibility of the devices used as measurement methods, or the fact that the costs are much higher than mobile applications, allow the use of mobile applications by sports scientists, strength and conditioning coaches, and practitioners [33]. Various parameters are measured by practitioners under physical conditions [3,34]. For example, it is used to measure balance [43], distance [44] and physical activity [45]. In addition, it has been reported in the research that the use of mobile applications increases the level of physical activity by increasing the level of physical fitness [46].

The systematization of evidence about the use of sports science apps was published in some recent systematic reviews [47,48,49]. However, no study (as far we know) has analyzed the validity and reliability of fitness and performance assessment apps. This is of paramount importance, since the inaccurate use of these systems when interpreting human performance could lead to inadequate decisions related to training design. In fact, if variation in performance is due to the inaccuracy or imprecision of the systems, the interpretation of results will not be appropriate.

For that reason, it is important to summarize the evidence regarding the validity and precision levels of sports science apps for measuring human strength, power, velocity, and change-of-direction capacities. Therefore, the purpose of this systematic review was two-fold: (1) to identify and summarize studies that have examined the validity of apps for measuring human strength, power, velocity, and change-of-direction, and (2) to identify and summarize studies that have examined the reliability of apps for measuring human strength, power, velocity, and change-of-direction.

## 2. Materials and Methods

The systematic review strategy was conducted according to PRISMA (Preferred Reporting Items for Systematic Reviews and Meta-analyses) guidelines [50]. The protocol was registered with the International Platform of Registered Systematic Review and Meta-Analysis Protocols with the number 202110089 and the DOI number 10.37766/inplasy2021.1.0089.

### 2.1. Eligibility Criteria

The inclusion and exclusion criteria can be found in Table 1.

The screening of the title, abstract and reference list of each study to locate potentially relevant studies was independently performed by the two authors. Additionally, they reviewed the full version of the included papers in detail to identify articles that met the selection criteria. An additional search within the reference lists of the included records was conducted to retrieve additional relevant studies. A discussion was made in the cases of discrepancies regarding the selection process with a third author (FMC and MRG). Possible errata for the included articles were considered.

### 2.2. Information Sources and Search

Electronic databases (Cochrane Library, PubMed, Scielo, and Web of Science) were searched for relevant publications prior to 16 January 2021. Keywords and synonyms were entered in various combinations in the title, abstract or keywords as follows: (“sport*” OR “exercise*” OR “athletic performance” OR “physical performance” OR “movement*”), AND (“mobile app*” OR “app*” OR “smartphone” OR “iphone”), AND (“Validity” OR “Accuracy” OR “Reliability” OR “Precision” OR “Varia*” OR “Repeatability” OR “Reproducibility” OR “Consistency” OR “noise”), AND (power OR velocity OR strength OR “change of direction”). Additionally, the reference lists of the studies retrieved were manually searched to identify potentially eligible studies not captured by the electronic searches. Finally, an external expert was contacted in order to verify the final list of references included in this scoping review in order to understand if there was any study that was not detected through our research. Possible errata were searched for each included study.

### 2.3. Data Extraction

A data extraction was prepared in Microsoft Excel sheet (Microsoft Corporation, Readmon, WA, USA) in accordance with the Cochrane Consumers and Communication Review Group’s data extraction template [51]. The Excel sheet was used to assess inclusion requirements, and subsequently tested for all selected studies. The process was independently conducted by the two authors. Any disagreement regarding study eligibility was resolved in a discussion. Full text articles excluded, with reasons, were recorded. All the records were stored in the sheet.

### 2.4. Data Items

The following information was extracted from the included original articles: (i) validity measure (e.g., typical error, absolute mean error, correlation coefficient); and (ii) reliability measure (e.g., intraclass correlation coefficient [ICC] and/or typical error of measurement [TEM] (%) and/or coefficient of variation [CV] (%) and/or standard error of measurement [SEM]). Additionally, the following data items were extracted: (i) type of study design, number of participants (n), age-group (youth, adults or both), sex (men, women or both), training level (untrained, trained); (ii) characteristics of the apps and comparator (for the case of validity studies); (iii) characteristics of the experimental approach to the problem, procedures and settings of each study.

### 2.5. Methodological Assessment

Two authors performed the methodological assessment of the studies eligible for inclusion using an adapted version of the STROBE assessment criteria, as was applied in O’Reilly et al. [52]. Hence, each article was evaluated using 10 specific criteria. If any disagreement appeared, it was discussed and solved by a consensus decision. The study rating was qualitatively interpreted following O’Reilly et al. [52]: from 0 to 7 scores, the study was considered as risk of bias (low quality), while, if the study was rated from 7 to 10 points, it was considered as a low risk of bias (high quality).

## 3. Results

### 3.1. Study Identification and Selection

**Figure 1:**
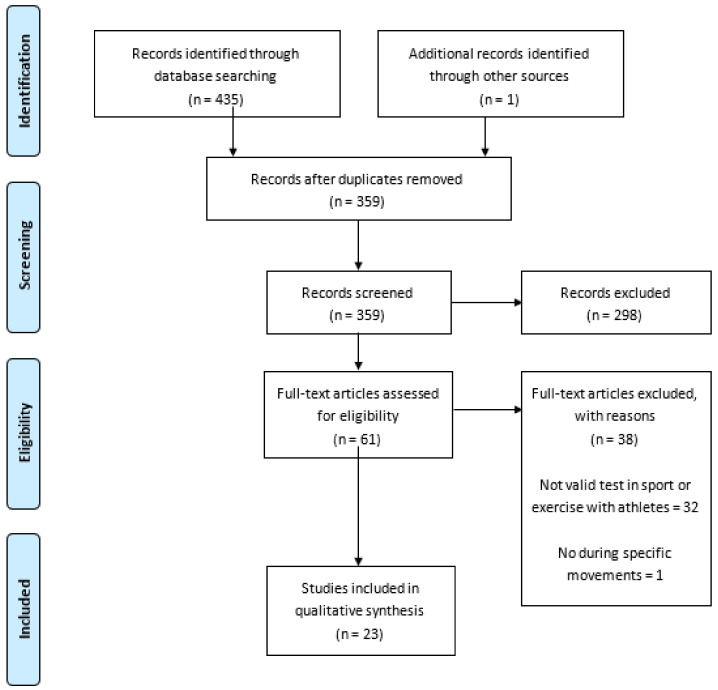
PRISMA flow diagram.

The searching of databases identified a total of 435 titles (Cochrane = 117; PubMed = 108; Scielo = 70; Web of Sciences = 140). In addition, one article was added from external sources. These studies were then exported to reference manager software (EndNote^TM^ X9, Clarivate Analytics, Philadelphia, PA, USA). The selection process can be observed in Figure 1.

### 3.2. Methodological Quality

The overall methodological quality of the cross-sectional studies can be found in Table 2.

### 3.3. Characteristics of Individual Studies

Characteristics of the included studies can be found in Table 3. The apps presented in the included articles were compared with motion a capture system [40,61,68,69], linear encoder and transducers [56,59,60,62,63,64,71], as well as with contact platforms [37,39,41,57,61,62,63,66], accelerometers [59,62,63,68,70], and time photocells [55,65,67,70].

Among the included studies, [39,53,56,59,62,63,64] tested the bench press, [64,68] the back squat, [60] the half squat and full squat [39,53], [40] the snatch, [53] the hip thrust, [68] the power clean, [37,41,57,61,66,71] the vertical jump (CMJ, SJ or DJ), [58] the running, [65,67] the sprint or agility [55,70] the static and dynamic arm swing.

Overall, 11 different apps were tested, in which [15,19,28,32,37,42] studies were conducted using the My Jump and My Jump App 2, [17,18,27,30,34,38,44,46] the Powerlift, previously named Mylift, [36] the Ergo Arm Meter, [35] the Smartphone accelerometer, [33,41] the Speedclock App, [31] the MySprint App, [29,45] the ILoad, and [26] the Styrd App.

### 3.4. Results of Individual Studies: Validity of Mobile Applications

Information of the validity levels obtained in the included studies can be found in Table 4. For the My Jump App and My Jump App 2, the correlation coefficient values of validity were between 0.926 and 9.995 [15,28,32,37,42]. For PowerLift and My Lift, the Pearson r values were r = 0.729–0.964 [18,30,34,45]. For the Ergo Arm Meter, the Pearson r value was r = 0.999 [36]. For the Smartphone Accelerometer, the Pearson r values were r = 0.54–0.93 [35,41]. For the Speedlock App, the Pearson r value was r = 0.93 [33]. For the MySprint App, the SEE values were from 0.007–0.015 m·s^−1^, and the Pearson r values from r = 0.989–0.999 [31]. For the ILoad App, the SEE values were from 0.003–0.004 m·s^−1^, and Pearson r values were r = 0.98–0.99 [29,47]. For the Styrd App, the SEE value was <7.3% and Pearson r value was r = 0.911. Finally, for the CODtimer App, the SEE value was 0.03s and Pearson r value was r = 0.998.

### 3.5. Results of Individual Studies: Reliability of Mobile Applications

Information on the reliability levels obtained in the included studies can be found in Table 5. For the My Jump App and My Jump App 2, the ICC values of reliability were from 0.492–0.999 and CV values were between 3.4% and 12% [15,19,32,37,42]. For the PowerLift and My Lift App, the ICC values of reliability were 0.70–0.989 [17,18,27,30,44,45] and CV values were between 3.97% and 10.4% [17,27,30,34,44]. For the Ergo Arm Meter, the SEM value of reliability was <13.1º/s [36]. For the Smartphone accelerometer, the ICC values of reliability were 0.634–0.99 [35,41]. For the MySprint App, the ICC value of reliability was 1 and CV values were from 0.027–0.14% [31]. For the ILoad App, the ICC value of reliability was 0.941 [47] and CV values were between 5.61% and 9.79% [29]. For the Styrd App, the ICC value was ≥0.980, the CV value was ≥4.3% and SEM was 12.5 w. Finally, for the CODtimer App, the ICC values of reliability range was 0.671–0.840, and CV values were between 2.2% and 3.2%.

## 4. Discussion

The need to assess and monitor the physical and performance status of athletes has led sports professionals to use equipment that might not be available in some sports and health contexts. Therefore, the use of mobile apps for these purposes has been gaining interest among the sports and scientific communities. However, coaches need to be confident that these apps measure what they are supposed to measure, and that their measurements are consistent and repeatable over time.

From the 24 included articles, both validity and reliability were tested for 11 different apps. However, one of the articles [60] tested only reliability. This discussion is organized based on the aims of assessing each app, considering the different models used for the same measures.

### 4.1. Validity of Mobile Applications

For the My Jump App and My Jump App 2, the correlation coefficient values of validity were between 0.926 and 9.995 [15,28,32,37,42]. For the PowerLift and My Lift, the Pearson r values were r = 0.729–0.964 [18,30,34,45]. For the Ergo Arm Meter, the Pearson r value was r = 0.999 [36]. For the Smartphone Accelerometer, the Pearson r values were from r = 0.54–0.93 [35,41]. For the Speedlock App, the Pearson r value was r = 0.93 [33]. For the MySprint App, the SEE values were between 0.007 and 0.015 m·s^−1^, and the Pearson r values were r = 0.989–0.999 [31]. For the ILoad App, the SEE values were between 0.003 and 0.004 m·s^−1^, and the Pearson r values were from r = 0.98–0.99 [29,47]. For the Styrd App, the SEE value was <7.3%, and the Pearson r value was r = 0.911. Finally, for the CODtimer App, the SEE value was 0.03s, and the Pearson r value was r = 0.998.

#### 4.1.1. Strength Apps

According to this systematic review, the Power Lift/My Lift app (which are the same) seems to be the most often used mobile app for assessing the strength status of humans. Furthermore, the studies revealed that, overall, the My Lift app is a valid tool for measuring displacement and velocity data based on different strength-based exercises. Thompson et al. [68] compared linear position transducers (LPTs), inertial measurement units (IMUs), and the My Lift app using an iPhone 7 with a 3D capture system that records time displacement data. The authors found that the LPT system had the greatest validity, and that the My Lift app’s validity (r ≥ 0.88) was similar to that of the LPT.

However, when using the My Lift app, the recorded data were limited to mean velocities [68]. Similarly, another study compared the My Lift app with a 3D capture system and found strong to very strong correlations between them for peak forward, backward, and vertical displacements, suggesting that the app is valid [40]. Furthermore, in contrast with the study of Thompson et al. [68], the peak vertical velocity from the My Lift app was analyzed, and had the greatest correlation with the gold standard equipment (r = 0.902), although it also had a higher standard error (SEE = 0.124 m.s^−1^) than the other displacement measures [40].

Interestingly, Courel-Ibáñez et al. [59] revealed a linear relationship (r = 0.939–0.920) between velocity outcomes derived from the My Lift app and a linear velocity transducer (LVT), considered by the authors as the gold standard device. However, the app produced absolute mean errors of 29.6% and 27.7% 1RM, and SEEs of 0.117 m.s^−1^ and 0.08 m.s^−1^ for bench press and back squat exercises, respectively. In fact, the same authors [59] suggested that the use of Pearson’s correlation coefficients might not be appropriate for analyzing the validity outcomes of a device, especially for devices that measure sensitive variables, such as bar velocity.

Notwithstanding the fact that, overall, the studies revealed acceptable validity of the My Lift app for measuring different displacement velocities for different exercises, most of the studies compared the My Lift app with different “reference” devices. In fact, while some authors refer to 3D capture systems as the gold standard device for velocity-based training (VBT) [40,68], others refer to LPTs as the gold standard [56]. Indeed, other studies noted that there is no evidence supporting the use of a 3D system as a reference device [59]. Therefore, more homogeneous study methodologies are needed for ensuring the veracity of such findings regarding the validity of the My Lift app.

In addition, two of the included studies tested the validity of the iLoad app [60,64]. Both studies compared the iLoad app with two different linear transducer systems. Despite the methodological differences between these two studies, the authors suggested that the app is a valid tool for measuring mean velocity during lower and upper body exercises. However, coaches need to manually manipulate the iLoad app when the exercise starts and stops, which may generate biological-based errors.

Furthermore, two other studies used basic smartphone accelerometer data to assess the mean bar velocity of different strength exercises [56,69]. The study of Viecelli et al. [69] revealed that the accelerometer app had a strong correlation (r > 0.93; *p* < 0.05) and a small absolute mean error (0.16%) when compared to a video recording system. Conversely, the other study [56] compared the accelerometer app with an LPT, revealing a lower correlation with the “reference” device (r = 0.54) than Viecelli et al. [69]. Moreover, the authors suggested that the app may not be completely valid for measuring strength because meaningful differences were found in mean velocities with higher lifting loads >90% 1RM [56].

A relevant issue regarding the studies that analyzed the validity of strength apps is the fact that some of them used Smith machines to try to eliminate horizontal bar displacements during exercises [62,72], while others used free-weight-based exercises [40,68]. As such, one can argue that lower bias is expected in studies using fixed-bar exercises when compared to those using free weights. Therefore, professionals using VBT should rely on the validity of devices that were tested in a similar apparatus than they will be using with their clients or athletes. Overall, the My Lift app seems to be the most often studied and valid option for measuring human strength.

#### 4.1.2. Power Apps

Of the studies included in this systematic review, three tested the validity of the My Jump app [41,61,66] and two tested the validity of the My Jump 2 app for analyzing jump height [57] and reactive strength index (RSI) measures [37]. The validity of the My Jump app, or measuring CMJ jump height, was tested using an iPhone 5s. Good accuracy (r = 0.995, *p* < 0.001) and a mean absolute error of 1.1–1.3 cm were recorded when compared with a force platform that was considered the “gold standard” device [41]. Another study that compared the same app on an iPhone 6 with a contact platform revealed almost perfect correlations for height measures of CMJ, SJ, and DJ (from a 40 cm box), with a standard error of 0.1 cm for all slow and fast stretch shortening cycle jumps [61]. Further, Stanton et al. [66] revealed that the My Jump app had a strong correlation (r > 0.99, *p* < 0.001) with a force plate for both CMJ and DJ. Moreover, the study that tested the validity of My Jump 2, regarding jump height, revealed the app’s validity (r = 0.98) when compared to a force platform and when compared to a yardstick apparatus [57].

When analyzing peak power using the My Jump app, an almost perfect correlation (r = 0.926) was found between the app and the Vertec jump system [71]. However, that same study showed a lower correlation (r = 0.813) when analyzing jump height [71]. This finding contrasts with the overall results of studies that revealed relatively high correlation values for jump height. In the study that analyzed the RSI measure using the My Jump 2 app, near-perfect correlations were found between the app and a force platform for the RSI values obtained for the 20 cm (r = 0.938) and 40 cm (r = 0.969) DJ heights [37]. However, the peak power measure revealed weak correlations for the 20 cm (r = 0.655) and 40 cm (r = 0.571) heights.

In summary, the My Jump and My Jump 2 apps are considered valid tools for assessing the vertical height and reactive strength index from different jump protocols using CMJ, SJ, and DJ. However, peak power assessments might not be as accurate as jump height assessments.

#### 4.1.3. Velocity Apps

Regarding running performance, three different apps were included in the present systematic review [58,65,67]. The MySprint app was compared to timing photocells and a radar gun to test its validity [65]. The results suggested that the app is valid, as near-perfect correlations were recorded between the app and the timing photocells for 40-m sprint splits (standard error = 0.007–0.015 s). Further, the My Sprint app showed almost perfect correlations with the radar gun for measures of the power, force, velocity, and mechanical properties of sprint performance [65,73]. However, the app needs to be manually manipulated to select the frames from the video recording, which can create a gap between the accuracy and error of the app.

The SpeedClock app showed excellent agreement when compared to timing lights, revealing a slight bias between the two devices [67]. Although the SpeedClock app was determined to be a valid tool, this finding is based only on a 10-m flying sprint. Thus, the validity of this app for measuring sprint running performance above 10 m remains unknown. An issue that must be addressed is the fact that these apps are accessible on different smartphone brands and models which record videos at varying frames per second, which could influence the accuracy and systematic bias of such apps. As such, it could be difficult to compare studies that test the validity of mobile apps for measuring running performance. Moreover, few studies have confirmed the validity of such apps in specific populations (e.g., athletes who participate in specific sports).

Furthermore, as the Stryd app assesses running power output, we have added this app to the velocity apps section [58]. The mentioned study tested and confirmed the validity of the app. The authors revealed that the power output measured by the Stryd app had strong associations (r = 0.911) with VO2max, which was obtained in a running-based incremental test. However, a standard error of 7.3% was found [58]. The same study also revealed that the Stryd app has the benefit of being connected with a sport watch. The literature is scarce regarding measures of power output using the Stryd app. For these reasons, future studies should rely on expanding this app’s validity to other populations and different methodologies, as the mentioned study included a small sample of only 12 male endurance athletes.

Despite the scarcity of studies on the validity of running-based apps, all apps that have been analyzed in such studies have been considered valid for the measures of movement displacement, velocity, time, and power output. Nevertheless, the standard errors of such apps must be carefully considered, as the user must manipulate the apps manually, which could increase the probability of human errors, especially when velocity is being measured.

#### 4.1.4. Change-of-Direction Apps

Of studies included in the present systematic review, only one tested the validity of a mobile app for measuring change-of-direction performance [55]. It showed that the CODtimer app had a very high correlation (r = 0.998) and a standard error of only 0.03 s regarding the timing gates for measuring change-of-direction total time [55]. Although that study showed the validity of the app, the authors suggested that the app might not be valid for change-of-direction tests that were not used in their study. For those reasons, future studies using the CODtimer app based on different change-of-direction tests are needed to ensure the validity of the app in different situations.

### 4.2. Reliability of Mobile Applications

For the My Jump App and My Jump App 2, the ICC values of reliability were 0.492–0.999 and CV values were between 3.4% and 12% [15,19,32,37,42]. For the PowerLift and My Lift App, the ICC values of reliability were 0.70–0.989 [17,18,27,30,44,45] and CV values were between 3.97% and 10.4% [17,27,30,34,44]. For the Ergo Arm Meter, the SEM value of reliability was <13.1º/s [36]. For the Smartphone accelerometer, the ICC values of reliability were between 0.634 and 0.99 [35,41]. For the MySprint App, the ICC value of reliability was 1 and CV values were from 0.027–0.14% [31]. For the ILoad App, the ICC values of reliability was 0.941 [47] and CV values were between 5.61% and 9.79% [29]. For the Styrd App, the ICC value was ≥0.980, CV value was ≥4.3%, and SEM was 12.5 w. Finally, for the CODtimer App, the ICC values of reliability range was 0.671–0.840, and CV values were between 2.2% and 3.2%.

#### 4.2.1. Strength Apps

Comparisons between the Power Lift/My Lift app and an LPT, a 3D motion capture system, and a 3-axis accelerometer, gyroscope, and magnetometer showed ICC values of up to 0.989 for the measures of bar mean velocity, peak vertical velocity, and peak forward and backward displacements [40,53,54]. However, none of these three studies included any information regarding the error of measurements or coefficients of variation of app measurements. Other studies [39,59] that also compared the app with diverse LPTs revealed that, even though the My Lift app presented ICC values between 0.973 and 0.993, the coefficient of variation ranged from 5.02% to 10.4%. The authors of those two studies [39,59] did not recommend using this app due to their substantial systematic bias. Conversely, Pérez-Castilla et al. [62] found small systematic bias and lower ICC values (0.70) than the abovementioned studies. Moreover, when measuring bar velocity, Thompson et al. [68] found coefficients of variation of <10% (for loads up to 70% of 1RM) and >10% (for loads above 90% of 1RM).

Furthermore, only one of the included studies tested the reliability of the iLoad app [64]. In line with the abovementioned study of Thompson et al. [68] regarding the My Lift app, the study of Pérez-Castilla et al. [64] revealed the acceptable reliability of the iLoad app when measuring bar velocity at lower 1RM percentages (the coefficients of variation ranged from 5.61% to 9.79%). Thus, when 1RM percentages were higher, the coefficient of variation values exceeded 10%, and the same pattern with similar values was found for the LPT system that the authors used in the same study [64]. For these reasons, professionals must be careful when using the iLoad app to measure bar velocity when heavier loads are involved, as the data extracted may be misleading. Moreover, using basic accelerometer data from a smartphone seems to have acceptable reliability [56,69]. Once more, it was found that, although the accelerometer app presented good agreement with an LPT, greater differences in mean bar velocity were, once again, found with heavier loads.

Velocity-based training (VBT) has been a topic of great interest given its practicability and ease of use. The most common equipment used for VBT seems to be LPTs and IMUs. However, these devices are expensive, and mobile apps are a potential affordable, valid, and reliable alternative. However, despite smartphone apps’ ability to measure bar velocity with good validity, they show greater systematic bias than gold standard measures, especially considering that the user is required to manually select the frames of video recordings.

#### 4.2.2. Power Apps

The reliability of the My Jump app was tested. After analyzing five CMJs, an almost perfect agreement was found (ICC = 0.999), presenting coefficients of variation of 3.4–3.6% for jump height using an iPhone 5s [41]. Another study also found an almost perfect agreement (ICC = 0.97–0.99) for DJ (from a 40 cm box), SJ, and CMJ heights when compared to a contact platform (coefficients of variation ranged between 3.8% and 7.6%) [61]. Stanton et al. [66] reported ICC values of 0.997 for CMJ and 0.998 for DJ heights. However, between-days systematic bias was detected for both CMJ and DJ mean values when the My Jump app was compared with a force platform. However, the same authors [66] revealed that the force plate showed lower values than the app at CMJ higher jump heights, and higher values at lower jump heights. As for DJ, the force plate produced higher values than the app at all jump heights [66].

Furthermore, when using the My Jump app to analyze peak power, only moderate ICC values were recorded for both males and females, with a wider confidence interval (CI) range calculated for males than females between poor and excellent ICC values [71]. The same study [71] revealed only poor absolute agreement for both males and females for the jump height measure. However, the authors compared the My Jump app with the Vertec system, which is not considered a gold standard for assessing power performance.

Regarding My Jump 2, two studies analyzed the reliability of the app for jump height and RSI measures [37,57]. My Jump 2 revealed acceptable intra-rater reliability for detecting changes in jump height measurements, with small variation detected between repeated tests [57]. Thus, the same study revealed that the app had moderate reliability (CV = 6.7%) when compared with the gold standard force platform. The other study that used the My Jump 2 app [37] also revealed near-perfect agreement between the app and a force platform for DJ jump height, contact time at 20 cm, and RSI measurements for 20 cm and 40 cm DJ heights. However, weak agreement was found for mean power. The RSI data extracted from the My Jump 2 app for 20 cm DJ had lower variation (CV = 6.7%) than the RSI data for higher DJ heights [37]. However, more studies need to be conducted on this new version of the My Jump app, as most of the studies focused only on the first version. The My Jump app has been found to be a reliable tool for measuring jump height.

#### 4.2.3. Velocity Apps

There is a lack of studies on the reliability of running-based velocity apps. In one such study that has been carried out, the MySprint app, a radar gun, and photocells yielded ICC values of 0.987 and 1 for mechanical variables and time measures, respectively [65]. Moreover, the same study revealed that the app produced a very low coefficient of variation in repeated trials (similar to the values found for the photocells and radar gun) for time and mechanical measures [65]. Similarly, the Stryd app revealed almost perfect ICC values (<10% coefficient of variation) when used to measure running power output in both indoor and outdoor situations. This highlights the benefits of this app for consistent use in various environments for measuring running performance. The use of the mentioned apps for measuring running-based velocity properties seems to be reliable, although more studies should be conducted to confirm this.

#### 4.2.4. Change-of-Direction Apps

The study of Balsalobre-Fernández et al. [55] revealed that the CODtimer app had near-perfect agreement with timing gates for measuring the total time in a change-of-direction test. The app presented similar ICC values (0.671–0.840) as timing gates for repeated trials and presented similarly low coefficients of variation (2.2% to 3.2%). Interestingly, the same study revealed that the app had moderate reliability for the left limb and good reliability for the right limb, resulting in similar limb asymmetry values between the app and the timing gates. Although there is a lack of studies regarding change-of-direction apps, the use of the CODtimer app can be an affordable choice for measuring change direction ability when expensive devices, such as timing gates or photocells, are not available.

### 4.3. Study Limitations, Future Research, and Practical Implications

Studies regarding the validity and reliability of mobile apps revealed some limitations that can be misleading. These limitations include (i) the limited sample sizes; (ii) the lack of studies regarding specific populations such as young athletes, adults, males, and females; (iii) the use of distinct testing protocols; (iv) the use of different smartphone brands and models in selected studies; and (v) greater focus on the validity and reliability of strength apps. Future studies should focus on analyzing the validity and reliability of such apps in specific populations with greater sample sizes. More consistent testing protocols and study methodologies must be conducted regarding the type of population, sample size, and smartphone brand and model.

Regarding the practical applications and the validity (Table 6) and reliability (Table 7) of the mobile applications, the My Jump and My Jump 2 apps, which are considered a video recorder with a 120-Hz high-speed camera, are valid tools for assessing reactive strength index, as well as movement displacements regarding vertical height, namely CMJ, SJ, and DJ. The Power Lift/My Lift app is considered a valid and reliable application for measuring peak velocity (vertical, horizontal, forward, and back displacement) frame by frame. The Ergo Arm Meter uses 3D data from a built-in accelerometer and gyroscope, and is considered a valid and accurate tool for measuring medium- to high-velocity movements of the arm in the sagittal plane. The smartphone accelerometer, which is a triaxial accelerometer, is considered a valid and reliable tool for assessing resistance exercise and peak vertical velocity. The Speedclock app, which records video at 60 frames per second, is considered a valid tool for measuring 10-m sprint performance [33]. However, the study that this is based on did not analyze this tool’s reliability.

The MySprint app, which records high-quality video at 240 frames per second, is a valid and reliable tool for assessing movement velocity, movement time, and power output. The iLoad app, which records linear and mean velocity, is also a valid and reliable tool for measuring workload and peak velocity. The Stryd app, which is a pedometer, is considered a valid and reliable tool for measuring movement displacement, power output, and workload. Finally, the CODtimer app, which records fill-HD video at a frequency of 240 frames per second, is considered a valid and reliable tool for measuring displacement regarding agility.

## 5. Conclusions

This systematic review revealed that MyLift App, PowerLift App, Smartphone with Mobile Basic Program Acelerometer, iLoad App, and MySprint app were valid and reliable for measuring movement velocity, while SpeedClock was only valid. For the case of the assessment of movement time, the CODtimer App and MySprint App were valid and reliable, while SpeedClock was valid and iLoad App was reliable. In the case the case of movement displacement, the CODtimer App, MyJump App, MyJump 2 App, Styrd App, ErgoArm meter were valid and reliable. For measuring power output, the Styrd App and MySprint App were valid and reliable. Finally, for monitoring workload, the Styrd App and iLoad App were considered valid and reliable.

## Figures and Tables

**Table 1:** Eligibility criteria.

Inclusion Criteria	Exclusion Criteria
Test of a mobile application in sport and exercise	Instruments other than mobile applications (e.g., computer software)
Tests were conducted in healthy athletes or recreationally healthy active adults for strength (e.g., resistance training exercises/movements), power (e.g., jumping, lifting movements), velocity (e.g., linear sprinting), and change-of-direction	The tests were not conducted in athletes (e.g., pregnant, elderly) or in healthy active adults (i.e., injury) for strength, power, velocity, and change-of-direction related movements (e.g., assessment of instruments without human action involved)
Estimation of movement velocity, movement time (e.g., a difference of time to complete a movement), and movement displacement (e.g., jump height)	Estimation of other outcomes than movement velocity, movement time, and movement displacement
In the case of validity, the apps were compared to the recognized gold standard: (1) Movement velocity (e.g., radar gun; isoinertial dynamometer consisting in cValid-extension linear position transducer; optoelectronic system) (2) Movement time (e.g., photocells) (3) Movement displacement (e.g., force plates, optoelectronic system)	For validity, the apps were not compared with recognized gold standard methods or were compared with other apps
In the case of validity, one of the following measures were included: (i) typical error; (ii) mean absolute error; (iii) correlation coefficient; and (iv) standard error of the estimate	For validity, outcomes presented are not typical error, mean absolute error, correlation coefficient or standard error of estimate.
In the case of reliability, one of the following measures were included: (i) intraclass correlation test; (ii) coefficient of variation; (iii) standardized typical error; and (iv) standard error of measurement.	For reliability, outcomes presented are not (i) intraclass correlation test; (ii) coefficient of variation; (iii) standardized typical error; and (iv) standard error of measurement.
Only original and full-text studies written in English	Written in language other than English. Other article types than original (e.g., reviews, letters to editors, trial registrations, proposals for protocols, editorials, book chapters and conference abstracts).

**Table 2:** Methodological assessment of the included studies.

Study	1	2	3	4	5	6	7	8	9	10	Quality
Balsalobre-Fernández et al. [40]	1	0	1	1	1	1	1	1	0	0	Low
Balsalobre-Fernández et al. [53]	1	0	1	1	1	1	1	0	0	0	Low
Balsalobre-Fernández et al. [54]	1	1	1	1	1	1	1	0	1	0	High
Balsalobre-Fernández et al. [55]	1	1	1	1	1	1	1	1	1	0	High
Balsalobre-Fernández et al. [41]	1	0	0	1	1	1	1	1	0	0	Low
Barrajón & Juan [56]	1	1	1	1	1	1	1	1	1	1	High
Brooks et al. [57]	1	1	1	1	1	1	1	0	1	1	High
Cerezuela-Espejo et al. [58]	1	1	1	1	1	1	1	1	0	1	High
Courel-Ibáñez et al. [59]	1	0	1	1	1	1	1	1	1	0	High
de Sá et al. [60]	1	1	1	1	1	1	1	1	1	1	High
Gallardo-Fuentes et al. [61]	1	1	1	1	1	1	1	1	1	1	High
Haynes et al. [37]	1	1	1	1	1	1	1	1	1	1	High
Martínez-Cava et al. [39]	1	0	1	1	1	1	1	1	0	1	High
Pérez-Castilla et al. [62]	1	1	0	1	1	1	1	0	0	1	Low
Pérez-Castilla et al. [63]	1	1	1	1	1	1	1	1	1	1	High
Pérez-Castilla et al. [64]	1	1	1	1	1	1	1	1	1	1	High
Romero-Franco et al. [65]	1	0	1	1	1	1	1	1	0	0	Low
Stanton et al. [66]	1	0	1	1	1	1	1	1	1	1	High
Stanton et al. [67]	1	0	1	1	1	1	1	1	1	1	High
Thompson et al. [68]	1	1	1	1	1	1	1	1	1	1	High
Viecelli et al. [69]	1	1	1	1	1	1	1	1	1	1	High
Yang et al. [70]	1	0	1	1	1	1	1	1	0	0	Low
Yingling et al. [71]	1	1	0	1	1	1	1	1	1	1	High

Note: provide in the abstract an informative and balanced summary of what was performed and what was found (item 1); state specific objectives, including any prespecified hypotheses (item 2); provide the eligibility criteria, and the sources and methods of selection of participants (item 3); for each variable of interest, offer sources of data and details of methods of assessment (measurement). Describe comparability of assessment methods if there is more than one group (item 4); explain how quantitative variables were handled in the analyses. If applicable, describe which groupings were chosen and why (item 5); give characteristics of study participants (item 6); summarize key results with reference to study objectives (item 7); discuss limitations of the study, considering sources of potential bias or imprecision. Discuss both direction and magnitude of any potential bias (item 8); give a cautious overall interpretation of results considering objectives, limitations, multiplicity of analyses, results from similar studies, and other relevant evidence (item 9); provide the source of funding and the role of the funders for the present study and, if applicable, for the original study on which the present article is based (item 10).

**Table 3:** Study characteristics.

Study	Outcome Tested	Tested Validity	Tested Reliability	App	App Characteristics	Comparator Characteristics	N/Population	Sex	Age	Experimental Protocol	Test or Movement	Validity Outcomes	Reliability Outcomes
Balsalobre-Fernández et al. [40]	Peak forward displacement; Peak backward displacement; Peak vertical velocity	Yes	Yes	My Lift App	Designed to automatically detect barbell trajectory	Vicon 3D motion capture system at 100 Hz (T-Series Cameras, Vicon Denver, Centennial, CO, USA).	10 Collegiate NCAA division I athletes	Male	20.9 ± 1.6 y.o.	Two repetitions with 40, 50, 60, 70 & 80% of their snatch 1-RM	Snatch	SEE; Cross Correlation coefficients Bland-Altman plots	ICC
Balsalobre-Fernández et al. [53]	Peak vertical Velocity	Yes	Yes	PowerLift App	Measure barbell velocity by video-recording thanks to the high-speed camera	Beast Sensor 3-axis accelerometer, gyroscope and magnetometer that measures velocity at a sampling rate of 50 Hz.	10 powerlifters	Male	26.1 ± 3.9 y.o.	Two repetitions with the five initial sets (which corresponded approximately to 50, 60, 70, 80, and 90% of the 1-RM)	Bench Press Hip Thrust Full squat	r-Pearson Bland-Altman plots	ICC
Balsalobre-Fernández et al. [54]	Peak vertical Velocity	Yes	Yes	PowerLift App	Measure barbell velocity by video-recording thanks to the high-speed camera	Smartcoach Power Encoder (Smartcoach Europe, Stockholm, Sweden) at 1000 Hz.	10 resistance trained athletes	Male	26.5 ± 6.5 y.o.	5 sets on the bench-press exercise with loads ranging 75–100% of 1RM.	Bench Press	SEE	ICC, Alpha Cronbach; Paired Sample t-test and Bland-Altman plots
Balsalobre-Fernández et al. [55]	Agility	Yes	Yes	CODtimer App	Record frequency of 240 frames per second (fps) at a quality of FullHD (1920 × 1080 pixels).	Witty gate, Microgate, Bolzano, Italy (with a 150 m range and a precision of ±0.4 ms).	20 adolescent soccer players	Male	13.85 ± 1.34 y.o.	6 trials (3 trials with COD executed with the right lower limb and 3 trials with COD executed with the left lower limb).	5 + 5 COD test measurement	Linear Regression; r-Pearson SEE	ICC Sample paired t-test and Bland-Altman plots.
Balsalobre-Fernández et al. [41]	Vertical Jump	Yes	Yes	My Jump App	A videorecord (240 frames per second): Includes a 120 Hz high-speed camera).	Force platform: recorded data at a sampling frequency of 1000 Hz.	20 recreationally students	Male	22.1 ± 3.6 y.o.	Each participant performed five CMJs.	Countermovement Jump	Bivariate Pearson ICC	ICC Cronbach’s alpha and CV
Barrajón & Juan [56]	Peak vertical Velocity	Yes	Yes	Smartphone with Mobile Basic Program Acelerometer	lis3dh tri-axial accelerometer (STMicroelectronics, Geneva, Switzerland) at 50 Hz.	Speed4Lifts Linear Transducer (Madrid, Spain).	10 young and healthy person	Male	23.1 ± 2.5 y.o.	Three sets of one repetition with a load of 70% 1RM. Three sets of one. repetition at 90% 1RM. 1RM attempt.	Bench Press	r-Pearson	ICC and Cronbach’s Alpha
Brooks et al. [57]	Vertical Jump	Yes	Yes	My Jump 2 App	Videorecord (240 frames per second): Includes a 120 Hz high-speed camera).	AMTI AccuPower force platform (Advanced Mechanical Technology Inc., MA, USA) at 400 Hz.	26 subjects	14 Male and 12 Female	23.2 ± 3.4 y.o.	3 jumps per participant.	Jump and Reach vertical jump test	r-Pearson Standardized mean bias	Standardized typical error ICC
Cerezuela-Espejo et al. [58]	Running Power	Yes	Yes	Styrd App	Pedometer (Stryd Summit Powermeter, firmware 1.2; Stryd, Inc., Boulder, CO, USA.	RunScribe(RunScribe Plus V3, Scribe Labs, Inc., Half Moon Bay, CA, USA). Garmin Running Power(v1.6, Olathe, Kansas, USA). Polar Vantage V (firmware 3.1.7, Polar, OY, Kempele, Finland)	12 endurance-trained athletes	Male	25.7 ± 7.9 y.o	3 min of work and 4 min of rest (3:4 ratio)—9 km·h^−1^ with 1 km·h^−1^ increments; 10 km·h^−1^, with weighted vest; 10 km·h^−1^, with treadmill inclination modified: −6%, −3%, 1%, +3% and +6%.	Running	SEM Repeated-Measures ANOVA	ICC Linear Regression r-Pearson SEE
Courel-Ibáñez et al. [59]	Peak Vertical Velocity	Yes	Yes	PowerLiftApp	Mean Velocity by video-recording the lift at slow motion (240 fps, 1080p): 240 Hz	T-Force Dynamic Measurement SystemTM (Ergotech Consulting, Murcia, Spain): 1000 Hz. ChronojumpTM (Chronojump, Bar- celona, Spain): 500 Hz VelowinTM: 500 Hz PushTM Band (PUSH Inc., Toronto, Canada):200 Hz	17 resistance-trained	males	26.0 ± 3.6 y.o	Two repetitions against fixed loads of 20, 30, 40, 50, 60, 70 and 80 kg.	Bench Press	ICC	CCC SEM
de Sá et al. [60]	Peak vertical Velocity	Yes	No	iLoad App	Record mean velocity (v 1.0; ILoad Solutions, Brasilia, Brazil)	Linear Encoder (Chronojump, Barcelona, Spain): displacement-time data at 1000 Hz.	16 young individuals	4 Female 12 Male	29.5 ± 7.2 y.o.	1st session—10 repetition maximum (RM) load. 2nd session—3 sets of 10 repetitions 10RM load.	Half Squat	Independent Sample t-test; ES; r-Pearson and Bland Altman	N.D.
Gallardo-Fuentes et al. [61]	Vertical Jump	Yes	Yes	My Jump App	A videorecord (240 frames per second): Includes a 120 Hz high-speed camera).	Contact Platform (Ergotester, Globus, Cologne, Italy): high speed video camera (300 frames per second).	21 athletes	14 male and 7 female	22.1 ± 3.6 y.o.	Five squat jumps, five countermovement jumps and five 40 cm drop jumps	Squat Jump Countermovement jump Drop Jump	r-Pearson Cronbach Alpha; ICC and Bland-Altman plots	ICC
Haynes et al. [37]	Reactive Strength Index	Yes	Yes	My Jump 2 App	A videorecord (240 frames per second): Includes a 120 Hz high-speed camera).	Force Platform (FP8, Hurlab, Finland): force platform, with a sampling frequency of 1200 Hz,	14 athletes	Male	29.5 ± 9.9 y.o.	Three DJ onto a force platform. Drop heigh of 20 cm and 40 cm was used.	Drop Jump	r-Pearson; Cronbach alpha; CV	ICC Bland-Altman plots
Martínez-Cava et al. [39]	Peak vertical Velocity	No	Yes	My Lift App	Peak vertical and horizontal displacement, peak and mean vertical velocity, instantaneous velocity and time (60 Hz).	T-Force Dynamic Measurement System (Ergotech Consulting, Murcia, Spain): 1000 Hz; Speed4Lifts (v2.0, Speed4Lifts, Madrid, Spain): 100 Hz STT (STT system, Basque Country, Spain): 100 Hz.	15 individuals	Male	27.0 ± 3.8 y.o.	One repetition against eight fixed loads (25, 35, 45, 55, 65, 75, 85 and 95 kg) at maximal intended velocity.	Bench Press Full Squat		ICC CCC SEE r-Pearson SEM
Pérez-Castilla et al. [62]	Load Velocity	Yes	Yes	PowerLiftApp	Mean Velocity by video-recording the lift at slow motion (240 fps, 1080 p): 240 Hz	Linear velocity transducer (T-Force [v.2.28, T-Force System, Ergotech, Murcia, Spain]: 1000 Hz; Chronojump [v.1.6.2, Chronojump Boscosystem?, Barcelona, Spain]; Speed4Lift [v.4.1, Speed4Lift, Madrid, Spain]: 1000 Hz; Velowin [v.1.6.314, Velowin, DeporTeC, Murcia, Spain]: 500 Hz; PUSH band [v1.1.26, PUSHTH band, PUSH Inc., Toronto, Canada]: 200 Hz; Beast sensor [v.2.3.7, Beast sensor, Beast Technologies Srl., Brescia, Italy]): 50 Hz	11 individuals	Male	22.5 ± 1.9 y.o.	1st session: load was incremented by 10 to 1 kg until the 1RM load was reached. 2nd session: 3 repetitions against 5 incremental loads (45–55–65–75–85%1RM), followed by 1RM.	Bench Press	ES SEE Two-way repeated-measured ANOVA	r-Pearson SEE
Pérez-Castilla et al. [63]	Peak Vertical Velocity	Yes	Yes	PowerLift App	Mean Velocity by video-recording the lift at slow motion (240 fps, 1080 p): 240 Hz	Trio-OptiTrack (V120:Trio; OptiTrack, Natu- ralPoint, Inc.):120 Hz Linear velocity transducer (T-Force [v.2.28, T-Force System, Ergotech, Murcia, Spain]: 1000 Hz; Chronojump [v.1.6.2, Chronojump Boscosystem?, Barcelona, Spain]; Speed4Lift [v.4.1, Speed4Lift, Madrid, Spain]: 1000 Hz; Velowin [v.1.6.314, Velowin, DeporTeC, Murcia, Spain]: 500 Hz; PUSH band [v1.1.26, PUSHTH band, PUSH Inc., Toronto, Canada]: 200 Hz; Beast sensor [v.2.3.7, Beast sensor, Beast Technologies Srl., Brescia, Italy]): 50 Hz.	14 individuals	Male	22.96 ± 1.6 y.o.	1st session: One 1RM in the bench press exercise. 2nd session: 3 repetitions against 5 loads (45, 55, 65, 75, and 85% of 1RM	Bench Press	Bland-Altman r-Pearson	CV ICC
Pérez-Castilla et al. [64]	Velocity	Yes	Yes	iLoad App	Record Linear velocity	T-Force system; Ergotech, Muscia, Spain) calculated at a sampling rate of 1000 Hz.	20 Students	Male	23.0 ± 2.6 y.o.	2 Sessions: 10 repetitions against four loads (25–40–55–70% of the 1RM.	Back Squat Bench Press	Samples t-test Hedge’sES SEE r-Pearson	SEM Hedge’s ES CV
Romero-Franco et al. [65]	Sprint Performance	Yes	Yes	MySprint App	240 fps high-speed camera at a quality of 720p	Radar gun (Stalker ATS ProII; Applied Concepts, Plano, TX, USA): sampling rate of 46.875 Hz. Timing photocells (Microgate, Bolzano, Italy)	12 Sprinters	Male	21.4 ± 3.9 y.o.	6 maximal effort 40-m sprints, with 5-min rest between trials, on a synthetic outdoor track.	40 m Sprints	r-Pearson SEE	ICC Bland-Altman plots CV
Stanton et al. [66]	Vertical Jump	Yes	Yes	MyJump App	A videorecord (240 frames per second): Includes a 120 Hz high-speed camera).	AMTI BP400 800–2000 force plate (Advanced Mechanical Technology Inc, Watertown, MA) collected at 1000 Hz.	29 adults	19 Female 10 male	26.41 ± 5.36	Two attempts with a two minute passive rest between attempts.	Countermovement Jump Drop Jump	r-Pearson ICC	Bland and Altman plots ICC
Stanton et al. [67]	Sprint Performance	Yes	No	Speedclock App	Records video at 60 frames per second	Smart-Speed Pro timing lights (Fusion Sport, Coopers Plains, Australia)	24 active individuals	female	>18 y.o.	Four maximal effort 20m sprints.	20 m Sprint	Independent t-test; ICC Bland Altman plots	
Thompson et al. [68]	Peack Vertical Velocity	Yes	Yes	MyLift App	Manual frame-by-frame inspection of slow-motion video. 240 Hz (720 p video quality)	3D motion capture (Raptor, Motion Analysis Cooperation, Rohnert Park, CA, USA) sampling at 250 Hz. Push Band (inertial measurement unit)—3 axis accelerometer at 1000 Hz	10 weightlifters	Male	25.0 ± 5.6 y.o.	Incremental load from 40‒100% 1RM (10% increments) 3 repetitions for light loads (≤60%), 2 repetitions for moderate loads (70‒80%), and 1 repetition for heavy loads (≥90%),	Back Squat Power Clean	Least Products Regression	Typical Error CV
Viecelli et al. [69]	Resistance exercise	Yes	Yes	Smartphone	3-axis accelerometer BMI160 (Robert Bosch GmbH, Stuttgart, Germany: 400 Hz)	Sony HDR-CX900E (Sony, Tokio, Japan): 400 Hz vs. 50 Hz.	22 participants			Two sets of ten repetitions of their 60% one repetition maximum	Adductor, Abductor, Chest Press, Leg Curl, Leg Extension, Leg Press, Lower Back, Total Abdominal and Vertical Traction	Bland-Altman plots r-Pearson	ICC
Yang et al. [70]	Arm posture and movement	Yes	Yes	ErgoArmMeter	Three-dimensional data from the built-in accelerometer and gyroscope (20 Hz)	Optical tracking system (OTS) (Elite, 2002; version 2.8.4380; BTS, Milano, Italy) with a sampling frequency of 100 Hz.	10 subjects	3 female 7 male	Median age: 24.5 y.o.	(1) static arm postures at three inclination angles in two different planes; (2) dynamic arm swings in the sagittal plane at three different rates; and (3) two simulated work tasks: mail sorting, and hair drying with a blow dryer.	Static posture Dynamic arm swing Dynamic work tasks	r-Pearson Bland-Altman plot	RMSD
Yingling et al. [71]	Peak Power	No	Yes	MyJump App	A videorecord (240 frames per second): Includes a 120 Hz high-speed camera).	Vertec (JUMPUSA.com, Sunnyvale, CA, USA)	135 subjects	94 males 41 females	18–39 y.o.	Three maximal Sargent VJ with countermovement	Countermovement Jump		ICC

**Table 4:** Validity of apps for estimation of movement velocity, movement time and movement displacement.

Study	App	SEE	Typical Error	Absolute Mean Error	Correlation Coefficient	Evidence
Balsalobre-Fernández et al. [40]	My Lift app	PVD: 0.056 m·s^−1^ PFD: 0.029 m·s^−1^ PBD: 0.048 m·s^−1^ PVV: 0.124 m·s^−1^		PVD: 0.053 ± 0.044 0.019 PFD: 0.030 ± 0.022 0.008 PBD: 0.044 ± 0.034 0.012 PVV: 0.113 ± 0.086	r = 0.729–0.902, *p* < 0.001	Authors claim the validity of the app.
Balsalobre-Fernández et al. [53]	PowerLift App	Full Squat: 0.04 m·s^−1^ Bench Press: 0.05 m·s^−1^ Hip Thrust: 0.03 m·s^−1^		Full Squat: 0.005 ± 0.04 Bench Press: 0.01 ± 0.05 Hip Thrust: 0.02 ± 0.04	Full Squat (r = 0.986, *p* < 0.005) Bench Press (r = 0.973, *p* < 0.005) Hip Thrust (r = 0.982, *p* < 0.005)	Authors claim the validity of the app.
Balsalobre-Fernández et al. [54]	PowerLift App	0.03 s.; *p* < 0.001			r = 0.964, *p* < 0.001	Authors claim the validity of the app.
Balsalobre-Fernández et al. [55]	CODtimer App	0.03 s.; *p* < 0.001			r = 0.998; *p* < 0.001	Authors claim the validity of the Iphone app.
Balsalobre-Fernández et al. [41]	My Jump App			1.1 ± 0.5 cm; 1.3 ± 0.5 cm	r = 0.995, *p* < 0.001	Authors claim the accuracy of the app.
Barrajón & Juan [56]	Smartphone with Mobile Basic Program Acelerometer	0.13 m/s = 0.83			r = 0.54, *p* < 0.001	Authors claim the validity for mean propulsive velocities but not in lower velocity ranges.
Brooks et al. [57]	My Jump 2 App		T.E = 0.18	Platforce platform = 0.96 Yardstick = 0.23 cm	Platform force: r = 0.98 Yardstick: r = 0.94	Authors claim acceptable validity compared with both the force platform and yardstick.
Cerezuela-Espejo et al. [58]	Styrd App	SEE < 7.3%			r = 0.911	Authors claim the validity of the app.
Courel-Ibáñez et al. [59]	PowerLift App	+=0.08 m.s^−1^		>27.7% 1RM		Authors did not recommend the app given their substantial errors and uncertainty of the measurements
de Sá et al. [60]	iLoad App	≤0.003 m s^−1^			Total Work: r = 0.997, *p* < 0.005 Mean Velocity: r = 0.987, *p* < 0.005.	Authors claim the validity of the app.
Gallardo-Fuentes et al. [61]	My Jump App	SJ: 0.1 ± 1.1 cm CMJ: 0.1 ± 1.0 cm DJ: −0.1 ± 0.7 cm			SJ (r = 2 0.96–0.99, *p* < 0.001) CMJ (r = 0.97–0.99, *p* < 0.001) DJ (r = 0.97–0.99, *p* < 0.001)	Authors claim the validity of the app.
Haynes et al. [37]	My Jump 2 App				RSI 20 cm: r = 0.938, *p* < 0.001. RSI 40 cm: r = 0.969, *p* < 0.001. Jump Height 20 cm: r = 0.812, *p* < 0.001. Jump Height 40 cm: r = 0.959, *p* < 0.001. Contact Time 20 cm: r = 0.963, *p* < 0.001. Contact Time 40 cm: r = 0.981, *p* < 0.001. Mean Power 20 cm: r = 0.655, *p* < 0.001. Mean Power 40 cm: r = 0.571, *p* < 0.001.	Authors claim the validity of the app.
Martínez-Cava et al. [39]	My Lift App	Bench Press: 0.10 + −0.97 Full Squat: -0.14 ± 0.10 m·s^−1^				My Lift app showed the worst result with errors well above the acceptable levels.
Pérez-Castilla et al. [62]	PowerLift App	≤4.46 kg		5.77 ± 3.58	r ≥ 0.94, *p* < 0.05	Authors claim the acceptable and comparValid accuracy of the app.
Pérez-Castilla et al. [63]	PowerLift App			−0.04 ± 0.02 m.s^−1^	r = 0.994, *p* < 0.05	Authors claim that smartphone application could be used to obtain accurate velocity measurements for restricted linear movements.
Pérez-Castilla et al. [64]	iLoad App	Back Squat: ≤0.04 m.s^−1^ Bench Press: 0.06 m.s^−1^			Back Squat: r = 0.98, *p* < 0.001 Bench Press: r = 0.98, *p* < 0.001	Authors claim that the app can be confidently used to quantify mean velocity.
Romero-Franco et al. [65]	MySprint App	0.007–0.015 s			r = 0.989-0.999, *p* < 0.001	Authors claim the validity of the app.
Stanton et al. [66]	MyJump app		1.0 cm		r > 0.99, *p* < 0.001	Authors claim the validity of the app.
Stanton et al. [67]	Speedclock App	0.13 s			r = 0.93, *p* < 0.05	Authors claim the valid tool for the assessment of mean 10m sprint velocity.
Thompson et al. [68]	MyLift App		Mean Velocity: 0.05 m·s^−1^		r ≥ 0.88, *p* < 0.05	Authors claim that smartphone applications could be used to obtain velocity-based data, but inertial measurements units demonstrate poorer validity.
Viecelli et al. [69]	Smartphone Accelerometer			0.16%	r > 0.93, *p* < 0.05	Authors claim that data from smartphone accelerometer- derived resistance exercise can be used to validly extract
Yang et al. [70]	ErgoArmMeter			<9.5º/s	r = 0.999	Authors claim that application is a valid method to measure upper arm elevation under static and dynamic conditions.
Yingling et al. [71]	MyJump App				Peak Power: r = 0.926 Vertical jump height: r = 0.813	Authors recommend the use of the APP during repeated measures within-subject testing of individuals or groups.

PVD: peak velocity displacement; PFD: peak forward displacement; PBD: peak backward displacement; PVV: peak vertical velocity, CMJ: countermovement jump; SJ: squat jump; DJ: drop jump; SEE: standard error of the estimate; s: seconds; cm: centimeters; r = correlation coefficient; m·s^−1^: meter per second; RM: repetition maximum.

**Table 5:** Reliability of apps for estimation of movement velocity, movement time and movement displacement.

Study	App	Intraclass Correlation Coefficient [ICC]	Typical Error of Measurement [TEM] (%)	Coefficient of Variation [CV] (%)	Standard Error of Measurement [SEM]	Evidence
Balsalobre-Fernández et al. [40]	My Lift app	ICC = 0.760–0.941				Authors claim the reliability of the app.
Balsalobre-Fernández et al. [53]	PowerLift App	ICC = 0.928–0.989				Authors claim the reliability and accuracy of the app.
Balsalobre-Fernández et al. [54]	PowerLift App	ICC = 0.965				Authors claim the reliability of the app.
Balsalobre-Fernández et al. [55]	CODtimer App	ICC = 0.671–0.840		CV = 2.2–3.2%		Authors claim the reliability of the Iphone app.
Balsalobre-Fernández et al. [41]	My Jump App	ICC = 0.999		Observer 1: CV = 3.4%; Observer 2: CV = 3.6%		Authors claim the reliability of the app.
Barrajón & Juan [56]	Smartphone with Mobile Basic Program Acelerometer	ICC = 0.634				Authors claim the reliability for mean propulsive velocities but not in lower velocity ranges.
Brooks et al. [57]	My Jump 2 App	ICC = 0.99	0.02 (90% CI: 0.02–0.02; trivial)	Platform force: CV = 6.7% Yardstick: CV = 12%		Authors claim acceptable reliability compared with both the force platform and yardstick.
Cerezuela-Espejo et al. [58]	Styrd App	ICC ≥ 0.980		CV ≥ 4.3%	SEM = 12.5W	Authors claim the reliability of the app.
Courel-Ibáñez et al. [59]	PowerLift App	ICC = 0.973		CV = 10.4%	SEM = 0.08 m.s^−1^	Authors did not recommend the app given the substantial errors and uncertainty of the measurements
de Sá et al. [60]	iLoad App	ICC = 0.941				Authors did not analyze the reliability of the app.
Gallardo-Fuentes et al. [61]	My Jump App	ICC = 11 0.97–0.99)		CV = 3.8–7.6%		Authors claim the validity and reliability of the app.
Haynes et al. [37]	My Jump 2 App	20 cm for RSI (ICC = 0.95 40 cm for RSI (ICC = 0.98) jump height (ICC = 0.96) 20 cm for jump height (ICC = 0.80)		RSI at 20 cm (CV = 6.71%) and at 40cm (CV = 10.32%). CV value for the 40cm jump was unacceptable		Authors claim the reliability of the app measuring the DJ on 20 cm.
Martínez-Cava et al. [39]	My Lift App	Full Squat: ICC = 0.993 Bench Press: ICC = 0.972		Full Squat: CV = 5.02% Bench Press: CV = 7.04%	Full Squat: SEM = 0.08 m.s^−1^ Bench Press: SEM = 0.08 m.s^−1^	My Lift app showed the worst result, with errors well above the acceptable levels.
Pérez-Castilla et al. [62]	PowerLift App	ICC = 0.73		CV = 3.97%		No reliability test was performed in the study
Pérez-Castilla et al. [63]	PowerLift App	ICC = 0.70		CV = 3.97%		Authors claim that smartphone application could be used to obtain accurate velocity measurements for restricted linear movements.
Pérez-Castilla et al. [64]	iLoad App			CV Range: 5.61–9.79%		Authors claim that the app can be confidently used to quantify mean velocity.
Romero-Franco et al. [65]	MySprint App	ICC = 1.0		CV = 0.027–0.14%		Authors claim the valid and reliValid using the app.
Stanton et al. [66]	My Jump app	ICC values range from 0.997 for CMJ to 0.998 for DJ				Authors claim the valid and highly reliValid tool using the app.
Stanton et al. [67]	Speedclock App	ICC = 0.93				Authors did not analyze the reliability of the app.
Thompson et al. [68]	MyLift App		TEM = 0.05 m.s^−1^	CV = 9.7 m.s^−1^		Authors claim that smartphone applications could be used to obtain velocity-based data, but inertial measurement units demonstrate poorer reliability and validity.
Viecelli et al. [69]	Smartphone Accelerometer	ICC > 0.99				Authors claim that data from smartphone accelerometer derived resistance exercise can be used to validly and reliably extract
Yang et al. [70]	ErgoArmMeter				SEM < 13.1º/s	Authors claim that application is a valid method to measure upper arm elevation under static and dynamic conditions.
Yingling et al. [71]	MyJump App	Peak Power: males (ICC = 0.747) females (ICC = 0.748) Vertical jump height: males (ICC = 0.492) females (ICC = 0.469)				Authors recommend the use of the APP during repeated measures within-subject testing of individuals or groups.

SEM: standard error of measurement; ICC: intraclass correlation; CV: % coefficient of variation; RSI: reactive strength index.

**Table 6:** Summary of validity of different apps.

	MyLift App	PowerLift App	CODtimer App	My Jump App	My Jump 2 App	Styrd App	Smartphone with Mobile Basic Program Accelerometer	Ergo Arm Meter	iLoad App	MySprint App	Speedclock App
Validity											
Movement velocity	Valid	Valid	Not valid	Not valid	Not valid	Not valid	Valid	Not valid	Valid	Valid	Valid
Movement time	Not valid	Not valid	Valid	Not valid	Not valid	Not valid	Not valid	Not valid	Not valid	Valid	Valid
Movement displacement	Not valid	Not valid	Valid	Valid	Valid	Valid	Not valid	Valid	Not valid	Not valid	Not valid
Power output	Not valid	Not valid	Not valid	Not valid	Not valid	Valid	Not valid	Not valid	Not valid	Valid	Not valid
Workload	Not valid	Not valid	Not valid	Not valid	Not valid	Valid	Not valid	Not valid	Valid	Not valid	Not valid

**Table 7:** Summary of reliability of different apps.

	MyLift App	PowerLift App	CODtimer App	My Jump App	My Jump 2 App	Styrd App	Smartphone with Mobile Basic Program Acelerometer	Ergo Arm Meter	iLoad App	MySprint App	SpeedClock App
Reliability											
Movement velocity	Reliable	Reliable	Not reliable	Not reliable	Not reliable	Not reliable	Reliable	Not reliable	Reliable	Reliable	Not tested
Movement time	Not reliable	Not reliable	Reliable	Not reliable	Not reliable	Not reliable	Not reliable	Not reliable	Reliable	Reliable	Not tested
Movement displacement	Not reliable	Not reliable	Reliable	Reliable	Reliable	Reliable	Not reliable	Reliable	Not reliable	Not reliable	Not tested
Power output	Not reliable	Not reliable	Not reliable	Not reliable	Not reliable	Reliable	Not reliable	Not reliable	Not reliable	Reliable	Not tested
Workload	Not reliable	Not reliable	Not reliable	Not reliable	Not reliable	Reliable	Not reliable	Not reliable	Reliable	Not reliable	Not tested
